# Paradoxical air embolism in patients undergoing hysteroscopic surgery for cesarean scar pregnancy: A case report and review of the literatures

**DOI:** 10.1002/ccr3.9060

**Published:** 2024-06-27

**Authors:** Fatemeh Amirkhanloo, Mohammad Haddadi, Mahbod Ebrahimi

**Affiliations:** ^1^ IVF Unit, Obstetrics and Gynecology Ward Yas Hospital Complex, Tehran University of Medical Sciences Tehran Iran; ^2^ Vali‐E‐Asr Reproductive Health Research Center, Family Health Research Institute Tehran University of Medical Sciences Tehran Iran

**Keywords:** air embolism, case report, cesarean scar pregnancy, hysteroscopy, women's health

## Abstract

Cesarean scar pregnancy cases who undergo hysteroscopic suction aspiration could be at higher risk of air emboli due to dilated, low‐resistant, high‐velocity blood vessels.

## 1 INTRODUCTION

Cesarean scar pregnancy (CSP) is a serious medical condition in which a blastocyst implants in a small tract on the uterine scar or in the dehiscence (niche) left behind by a previous cesarean incision. This condition can be life‐threatening, and its incidence has been gradually increasing worldwide. It is estimated to occur in approximately 1 in every 1800 to 1 in every 2226 overall pregnancies.^1^

Multiple strategies, alone or in combination, have been used in CSP management including expectant management, invasive or non‐invasive interventions, such as methotrexate injection, potassium chloride injection in the fetal heart, uterine artery embolization, hysterectomy, etc.^2^, ^3^; however, due to lack of robust standard clinical trials, the standard treatment is not clear.

Local methotrexate injection followed by suction aspiration and hysteroscopy for CSP removal has advantages such as fertility preservation and low bleeding rate.^4^ However, potentially life‐threatening air emboli (AE) have been described as a rare complication.^5^, ^6^, ^7^

Given air embolism is a rare complication of hysteroscopy procedures, the healthcare provider may not be aware of this potentially fatal complication. Consequently, in the present article, we described our experience with a case of paradoxical air embolism during hysteroscopy surgery for cesarean scar pregnancy.^8^ This case is written based on the SCARE checklist.^9^

## 2 CASE HISTORY

**FIGURE 1:**
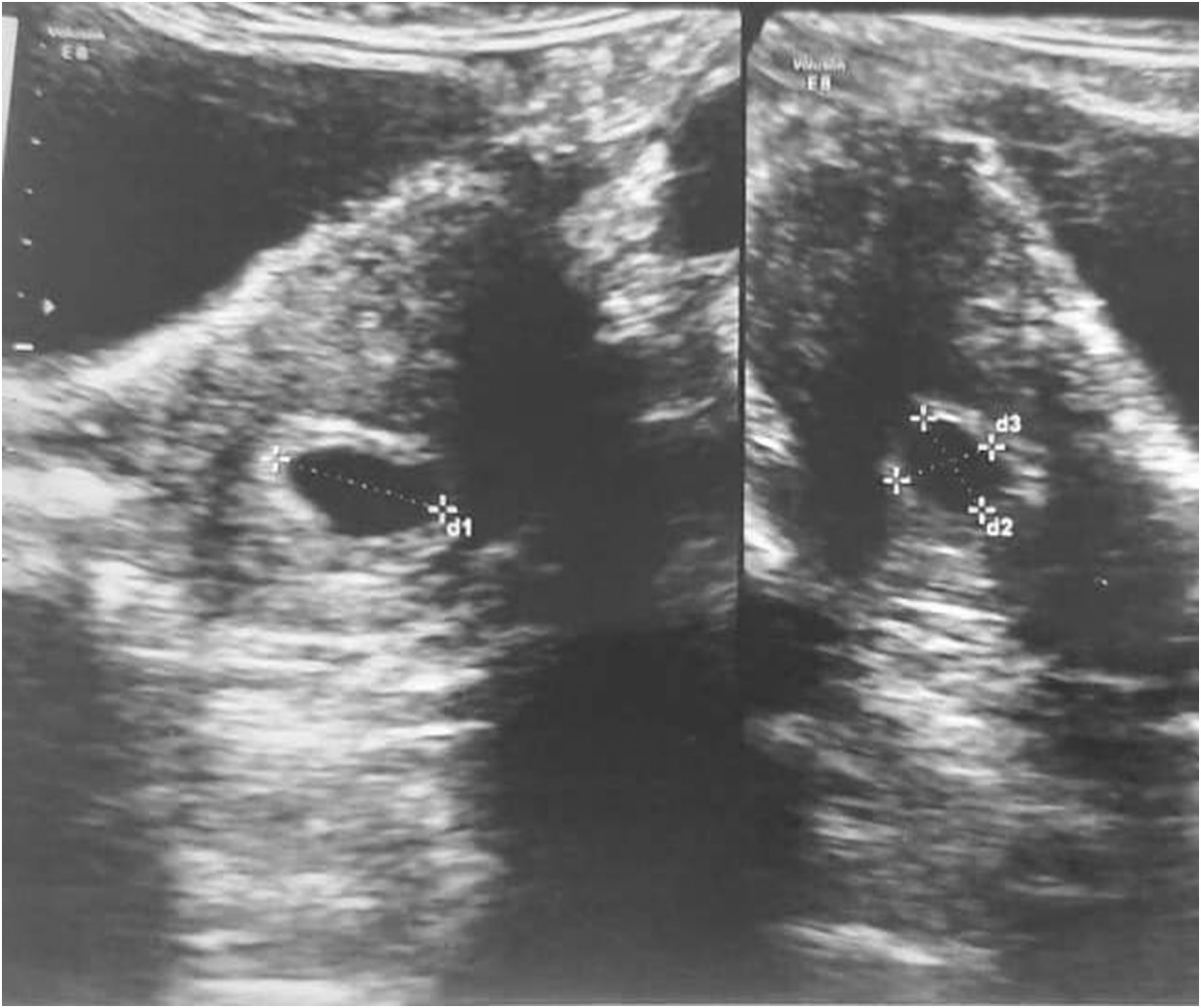
This abdominal ultrasonography shows a gestational sac containing a fetal pole.

A 30‐year‐old pregnant woman (G3P1Ab1L1) presented with mild general abdominal pain and spotting in the last 2 weeks. Her gestational age was 6 weeks and 5 days due to the last menstrual period. Prior abdominal ultrasonography showed a gestational sac containing a fetal pole with fetal heart rate (FHR) in the inferior segment of the uterus, expanding to the cesarean scar (Figure 1).

The first pregnancy was terminated by elective cesarean in 39 weeks + 3 days without any complications. The second pregnancy was aborted in the first trimester, and she underwent hysteroscopy due to the removal of remnant tissue after 2 weeks. Her past medical history was uneventful apart from receiving vitamin supplements and folic acid during the present pregnancy.

At admission, the vital signs were stable (temperature 37°C, respiratory rate = 14 breaths per minute, blood pressure = systolic: 117 mm Hg, diastolic =73 mm Hg, heart rate = 80 bpm, and SPO_2_ = 98%). In the physical exam, the abdomen exam was soft without any tenderness. A closed external OS with mild bleeding was detected in the vaginal exam. BHCG was 86,756 mIU/mL. Transvaginal sonography (TVS) at the hospital showed a gestation sac with CRL = 9.7 mm (equivalent to 7 weeks of gestation). Fetal heartbeat was present in the normal range and the sac was implanted in the niche. The thickness of the myometrium on the scar was 1.7 mm, and the ovaries had normal shape and texture. The first diagnosis was the C/S pregnancy in the niche.

## 3 METHODS

In the first step, an ultrasound‐guided injection of 50 mg of methotrexate was done under general anesthesia. The patient left the operating room (OR) in stable condition. After 24 h, TVS was performed, and the FHR was not present. After 48 h, the fetal heart was not seen and beta HCG was 74,874 mIU/mL. After discussing treatment options, she consented to suction aspiration and concomitant hysteroscopy removal of remnant tissues. In the operation room, she underwent spinal anesthesia, and standard noninvasive monitoring was used, in ultrasound, a gestational sac was seen with a fetal pole without fetal heart rate in the niche. The gestation sac in the niche was observed after dilating the cervix by Hegar. Then, the curettage tool was placed in line with the niche, and retained products of conception were removed with 200 mmHg of the suction pressure. The retained products of conception were still observed by ultrasonography in the scar location.

After the air bubbles existed through hysteroscopy tubes, the hysteroscopy was inserted into the cavity with a pressure of 80 mm Hg, with isotonic media (0.9% NaCl as the uterine distention). The retained conception of products was removed by pressure of the media. We used approximately 1000 cc of normal saline and the deficit was 100 cc. The cervix was covered by wet gauze after removing the hysteroscope. She developed respiratory distress (respiratory rate = 65, SPO_2_ = 38%), confusion, and peripheral cyanosis, approximately 5–6 min after the uneven operation.

First, mechanical ventilation with an oxygen mask was started but SPO_2_ was still decreasing. She underwent intubation. Capnography was started due to bradycardia (heart rate = 30 beats per min) and atropine and vasopressin were injected. Transthoracic echocardiographic showed many bubbles in the right ventricle and left atrium. Paradoxical air embolism was confirmed and treatment was instituted immediately.

A central venous catheter was placed in the internal jugular vein for air retrieval and the patient was put in a lateral decubitus position. A small amount of air was withdrawn. Approximately 25 min after the start of the event. Systolic and diastolic blood pressure, heart rate, SPO_2_, and ECO_2_ slowly recovered. Given the patient's stable hemodynamic and pulmonary condition on mechanical ventilation in the following 2 h, the decision was made to proceed to the intensive care unit.

## 4 CONCLUSION AND RESULTS

A brain computerized tomography (CT) scan was requested for her, which showed generalized brain edema and transtentorial herniation. After examination by a neurologist, brain death was diagnosed. Her family did not have consent to organ donation. She was observed in the ICU. Finally, cardiac arrest occurred for her after 13 days.

## 5 DISCUSSION

Multiple strategies, alone or in combination, have been used in CSP treatments, including expectant management, local or systemic methotrexate injection, Intragestational injection of KCl or Methotrexate (MTX), uterine artery embolization, hysteroscopy, ultrasound‐guided suction aspiration with or without local injection of vasopressin, transcervical insertion of the balloon catheter, laparoscopic hysterectomy, and hysterectomy^2^, ^3^, ^10^; however, the standard treatment is not clear.

Given the high risk of severe maternal morbidity, some experts prefer to apply intervention at a tertiary care hospital rather than expectant management.^4^, ^11^ Among the therapeutic modalities, the best method should be selected according to the patient medical condition, fetal viability, gestational age, the patient's desire for future pregnancy, and the available facilities, as well as the skill and experience of the surgeons.^2^

Ultrasound‐guided intragestational injection of MTX followed by suction aspiration and surgical hysteroscopy is one of the appropriate and beneficial treatments for CSP due to its ability for fertility reservation,^4^ while life‐threatening AE can be a rare complication in surgical hysteroscopy and suction aspiration.^5^, ^6^, ^7^ AE episodes could have lethal impacts and result in paradoxical embolism (in patients with venous‐to‐arterial communications or extra‐cardiac pathway or when it is more than the lung filtering capacity), and serious neurological and cardiac complications such as cardiac failure and cardiac arrest.^8^

There is no sufficient evidence of AE incidence following CSP suction aspiration and hysteroscopic removal of the conceptional product. However, a case‐series study detected only five (0.09%) incidents of VGE during hysteroscopic endometrial ablation.^12^ In our center, from 117 cases of CSP who underwent suction aspiration and hysteroscopic removal of the conceptional product, no case of AE has been detected (unpublished data). This patient was the first case of AE during operative procedures.

When it comes to surgical hysteroscopy, capnography can play a valuable role in quickly identifying and addressing AE incidents. Unfortunately, in our situation, the use of spinal anesthesia meant that capnography monitoring was not employed by the anesthesiologist. This resulted in a delayed diagnosis. To minimize complications and detect AE incidents early, it is recommended that general anesthesia be utilized in conjunction with capnography monitoring. Additionally, it is worth noting that severe vaginal bleeding is a major risk factor for emboli formation during hysteroscopy procedures. To mitigate this risk, local injection of vasopressin can help to decrease bleeding and minimize the chance of AE occurrence.^13^

In contrast, prompt therapeutic measures for a patient with AE entail halting the surgery without delay, repositioning the patient in a left lateral decubitus (upright) posture, administering 100% O_2_ ventilation support, providing intravenous fluids, and, in instances of significant embolism, central venous catheterization and circumventing the patient's cardiopulmonary system are advised.^12^, ^14^

Also, there is no published data in the literature about the difference in AE incidences following suction aspiration or hysteroscopy procedures in pregnant women and non‐pregnant ones.

In the first trimester of pregnancy, the syncytiotrophoblast layer produces a glycoprotein, HCG molecule. It is composed of two subunits termed α and β subunits. The β‐subunit (β‐HCG) is characterized by distinctly different amino‐acid sequences and its serum concentration is often used as the first diagnostic test to identify pregnancy.^15^

In addition, the syncytiotrophoblast layer facing the uterine circulation plays a critical role in the invasion of the maternal decidua basalis and spiral arteries and routes the blood flow through the placenta.^15^ Consequently, maternal serum concentrations of its products (e.g., β‐HCG) could be biochemical markers of the invasion to maternal circulation and occurrences of some surgical complications during suction aspiration and operative hysteroscopies such as vaginal bleeding and AE.

On the day of the operation, the presented case showed a serum plasma concentration of β‐HCG of 74,874 mIU/mL, indicating a well‐established vasculature around the gestational product. We recommend delaying surgical procedures until there is a significant decrease in serum (β‐HCG) levels as a marker of a reduction in blood supply around the gestational sac to minimize the risk of AE. Additionally, we suggest a cervical injection of vasopressin to reduce the absorption of distention media and prevent excessive blood loss and AE, but it should be performed with caution. Intravascular absorption of vasopressin could lead to bradycardia and hypertension.^15^

## AUTHOR CONTRIBUTIONS

**Fatemeh Amirkhanloo:** Data curation; project administration; writing – review and editing. **Mohammad Haddadi:** Writing – original draft; writing – review and editing. **Mahbod Ebrahimi:** Conceptualization; data curation; project administration; supervision.

## FUNDING INFORMATION

None.

## CONFLICT OF INTEREST STATEMENT

The authors report no conflict of interest.

## ETHICS STATEMENT

This case was approved by the ethical committee of the Tehran University of Medical Sciences on 1 September 2023. The patient signed the informed consent. This study was performed according to Helsinki declaration and the participant's identity is confidential.

## CONSENT

Written informed consent was obtained from the patient to publish this report in accordance with the journal's patient consent policy.

## Data Availability

The data that support the findings of this study are available from the corresponding author upon reasonable request.
